# The Role of Tissue Oxygen Tension in Dengue Virus Replication

**DOI:** 10.3390/cells7120241

**Published:** 2018-12-01

**Authors:** Efseveia Frakolaki, Panagiota Kaimou, Maria Moraiti, Katerina I. Kalliampakou, Kalliopi Karampetsou, Eleni Dotsika, Panagiotis Liakos, Dido Vassilacopoulou, Penelope Mavromara, Ralf Bartenschlager, Niki Vassilaki

**Affiliations:** 1 Laboratory of Molecular Virology, Hellenic Pasteur Institute (HPI), 11521 Athens, Greece; sevif@pasteur.gr (E.F.), yioula.kai27@gmail.com (P.K.); moraiti.biology@gmail.com (M.M.); e.kalliampakou@pasteur.gr (K.I.K.); 2 Laboratory of Cellular Immunology, Hellenic Pasteur Institute, 11521 Athens, Greece; karampetsou@pasteur.gr (K.K.); e.dotsika@pasteur.gr (E.D.); 3 Laboratory of Biochemistry, School of Medicine, University of Thessaly, 41500 Larissa, Greece; pliakos@med.uth.gr; 4 Section of Biochemistry and Molecular Biology, Faculty of Biology, National and Kapodistrian University of Athens, 15701 Athens, Greece; didovass@biol.uoa.gr; 5 Laboratory of Biochemistry and Molecular Virology, Department of Molecular Biology and Genetics, Democritus University of Thrace, 68100 Thrace, Greece; pmavrom@mbg.duth.gr; 6 Department of Infectious Diseases, Molecular Virology, University of Heidelberg, 69120 Heidelberg, Germany; Ralf.Bartenschlager@med.uni-heidelberg.de; 7 German Center for Infection Research, Heidelberg partner site, 69120 Heidelberg, Germany

**Keywords:** hypoxia, dengue virus, hepatocytes, HIF, AKT, metabolic reprogramming, glycolysis

## Abstract

Low oxygen tension exerts a profound effect on the replication of several DNA and RNA viruses. In vitro propagation of Dengue virus (DENV) has been conventionally studied under atmospheric oxygen levels despite that in vivo, the tissue microenvironment is hypoxic. Here, we compared the efficiency of DENV replication in liver cells, monocytes, and epithelial cells under hypoxic and normoxic conditions, investigated the ability of DENV to induce a hypoxia response and metabolic reprogramming and determined the underlying molecular mechanism. In DENV-infected cells, hypoxia had no effect on virus entry and RNA translation, but enhanced RNA replication. Overexpression and silencing approaches as well as chemical inhibition and energy substrate exchanging experiments showed that hypoxia-mediated enhancement of DENV replication depends on the activation of the key metabolic regulators hypoxia-inducible factors 1α/2α (HIF-1α/2α) and the serine/threonine kinase AKT. Enhanced RNA replication correlates directly with an increase in anaerobic glycolysis producing elevated ATP levels. Additionally, DENV activates HIF and anaerobic glycolysis markers. Finally, reactive oxygen species were shown to contribute, at least in part through HIF, both to the hypoxia-mediated increase of DENV replication and to virus-induced hypoxic reprogramming. These suggest that DENV manipulates hypoxia response and oxygen-dependent metabolic reprogramming for efficient viral replication.

## 1. Introduction

The dengue virus (DENV) is an important mosquito-borne member of the *Flavivirus* genus in the *Flaviviridae* family, causing widely distributed and endemic, visceral, and central nervous system diseases [1]. Symptoms of infection with any of the four DENV serotypes range from mild (dengue fever) to the more severe dengue hemorrhagic fever (DHF) and dengue shock syndrome (DSS) [2]. Secondary heterotypic infection is a risk factor to develop DHF/DSS, mediated most likely by antibody-dependent enhancement of infection (ADE) [3]. The global incidence of dengue has grown dramatically in recent decades [4,5,6]. Unfortunately, the recently approved dengue vaccine has only limited overall efficacy [7]. Moreover, there is no approved antiviral therapy [8].

The genome of DENV consists of a positive single-strand RNA of ~11 kb in length, composed of a 5´ untranslated region (UTR) with a m^7^G cap structure, a single open reading frame encoding for the viral polyprotein and a 3´ UTR [9,10]. The polyprotein is processed into structural proteins (C, prM, E) and non-structural (NS) proteins (NS1, NS2A, NS2B, NS3, NS4A, NS4B, NS5). The latter are involved in viral RNA replication via the synthesis of a negative-sense RNA intermediate, virus assembly, and modulation of host cell immune responses. During DENV replication in host cells, two types of programmed cell death are induced: apoptosis [11,12] and pyroptosis (osmotic lysis) [13,14]. DENV promotes apoptosis through downregulation of the Bcl-2-mediated PI3K/AKT signaling pathway [15]. However, at the early stage of infection the virus activates transiently PI3K signaling to block early apoptotic cell death, which enhances virus replication [16]. Moreover, through the use of a PDK1 inhibitor, it was shown that the PI3K/AKT pathway can regulate DENV infection by promoting cell survival as well as by contributing to virus entry and viral RNA translation [17].

DENV has a rather broad tissue tropism and was found to replicate in cells of different organs, such as hepatocytes, type II pneumocytes, cardiac fibers, tissue-resident and circulating monocytes/macrophages, and endothelial cells [18,19]. The liver is an important target organ for DENV that causes metabolic disturbances with varying degrees of injury, ranging from mildly raised transaminases to fulminant liver failure [20,21].

DENV replication and the activity of antiviral drugs in cultured cells have been traditionally studied under ambient oxygen tension (20% *v*/*v* O_2_) [12,15,16,17,22]. However, oxygen levels in most mammalian tissues, including the liver and monocytes, are substantially lower (1–11% O_2_) than atmospheric O_2_ levels [23]. This is an understudied, but important, aspect because low oxygen triggers an adaptive reprogramming towards anaerobic glycolysis [24] in many cells, including hepatocytes [25] and monocytes [26,27]. In addition, low oxygen levels corresponding to those in vivo have profound effects on the replication efficiency of many viruses as compared to culturing of the cells under atmospheric oxygen level [28]. We have previously established hepatocyte culture-based infection models adapted to low oxygen tensions simulating the physiological ones in the liver (3–12% O_2_) that turned out to favor RNA replication of the hepatitis C virus (HCV) belonging to the *Flaviviridae* family like DENV [25]. This enhancement was independent from hypoxia inducible factors (HIF)-1α and -2α and directly linked to an increase in anaerobic glycolysis as well as an upregulation of oncogenes associated with glucose metabolism (AKT, AP-1). Moreover, a report has shown that hypoxia (3% O_2_) enhances DENV entry into THP-1 monocytes under ADE conditions via HIF1α-dependent upregulation of the FccRIIA receptor as well as HIF1α-independent alterations in membrane ether lipid concentrations [29]. Non-ADE DENV infection was also reported to be enhanced under low oxygen conditions, however the underlying mechanism remains to be defined.

Based on these observations, we studied the impact of oxygen tension on DENV replication and virus production in liver cells, monocytes, and epithelial cells. We show that low oxygen selectively enhances an early step of DENV RNA replication correlating directly with increases in oxidative response and anaerobic glycolysis. Moreover, we provide evidence that DENV induces a hypoxic response and subsequent metabolic reprogramming, thus uncovering a bidirectional relationship between DENV and oxygen tension that is important for viral replication.

## 2. Materials and Methods

### 2.1. Cell Culture

Huh7 (Registry No. JCRB0403) [30] and Vero E6 cells (originally obtained from ATCC#CRL-1586) were cultured in high glucose (25 mM) Dulbecco’s modified minimal essential medium (Thermo Fisher Scientific, Waltham, Massachusetts, USA), supplemented with 2 mM L-glutamine, 0.1 mM non-essential amino acids, 100 U/mL penicillin, 100 µg/mL streptomycin, and 10% (*v*/*v*) fetal calf serum (referred to as complete DMEM). To create oxygen tensions lower than the atmospheric one, cells were cultured in a fully humidified incubator supplied with pure nitrogen gas to reduce oxygen as well as with 5% (*v*/*v*) CO_2_ at 37°C (New Brunswick CO_2_ incubator; Artisan Technology Group, Champaign, IL, USA) [31].

### 2.2. Viruses and Plasmid Constructs

**Figure 1:**
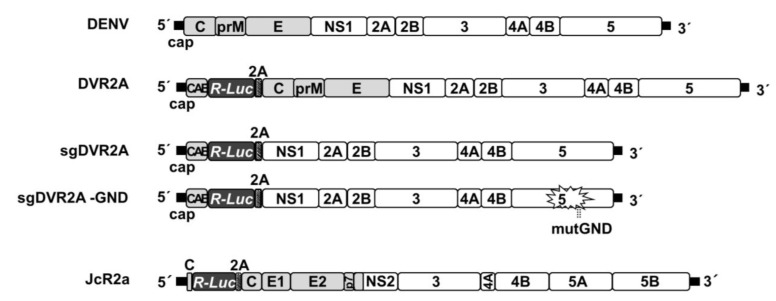
**Schematic representation of virus constructs used in this study.** From top to bottom: DENV2 full-length genome; DVR2A, derived from the DENV2 full-length genome by insertion of a *Renilla* luciferase (R-Luc) coding sequence downstream of the capsid cyclization sequence (CAE) and upstream of a *Tosea asigna* virus 2A protease cleavage site; sgDVR2A, a subgenomic reporter replicon derived from the DENV2 full-length genome by insertion of R-Luc coding sequence in-between CAE and the 2A cleavage site sequence. The last 24 amino acid residues of the envelope coding region (TM) at the N-terminus of NS1 ensure proper membrane topology of the polyprotein. sgDVR2A-GND, a replication-deficient NS5 mutant of sgDVR2A. All DENV constructs are derived from the DVs2 16681 isolate. JcR2a, a Jc1 (J6CF-JFH1 chimera) derivative containing the R-Luc gene fused N-terminally to 16 codons of the core gene (C) and C-terminally to the FMDV 2A protease cleavage site (grey striped box); Black bars in all panels indicate UTRs. Polyprotein cleavage products are labeled as specified in the introduction.

Plasmids carrying the full-length genomes pFK-DVs and pFK-DVR2A (with a *Renilla* luciferase reporter gene), as well as the subgenomic replicons pFK-sgDVR2A and pFK-sgDVR2A-GND (a replication-deficient NS5 mutant), are based on the DV-2 16681 strain and have been described previously [32,33]. Plasmid pFK-I389RLuc2ACore-3’-Jc1 (JcR2a), has been described previously [34]. Schematic representation of virus constructs is shown in Figure 1. HIF-1α and HIF-2α expressing plasmids, pEGFP-HIF-1α (kindly provided by G. Simos, University of Thessaly, Larissa, Greece) and pEGFP-HIF-2α, respectively, have been described previously [35,36]. p9×HRE-Luc carries nine copies of hypoxia response element (HRE) and rat prolactin minimal promoter upstream of the firefly luciferase gene (kindly provided by R. Hernandez-Alcoceba, University of Navarra, Pamplona, Spain) [37].

### 2.3. In Vitro Transcription 

Full-length and subgenomic DENV constructs were linearized with XbaI and used for in vitro transcription as described previously [32]. HCV constructs were linearized with MluI and used for in vitro transcription as described previously [38].

### 2.4. Transfection Assays

Electroporation with in vitro transcribed full-length viral RNAs into Vero E6 cells and bicistronic DENV RNAs into Huh7 cells was performed as described elsewhere [39]. For plasmid DNA and siRNA transfections, Huh7 cells seeded at 50–60% confluence were treated with Lipofectamine 2000 transfection reagent (Thermo Fisher Scientific, Waltham, MA, USA) as recommended by the manufacturer. The small interfering RNAs (siRNAs) targeting HIF-1α (5’-AGGAAGAACTATGAACATAAA-3’; NM-001530) and HIF-2α (5’-CCCGGATAGACTTATTGCCAA-3’; NM-001430) and the AllStars negative-control siRNA were obtained from Qiagen (Düsseldorf, Germany).

### 2.5. Preparation of Virus Stocks and Infection Assays

DENV virus stocks were generated in Vero E6 cells as described elsewhere [32] and used to inoculate cells for 4 h, unless otherwise specified. For DVR2A infectivity assays, supernatants from the first round of infection were used to infect naïve cells. HCV virus stocks were generated as described elsewhere [39] and used to infect naive Huh7.5 cells.

### 2.6. Virus Titration in Cell Culture Supernatants

DENV virus titers were determined by standard plaque assay (PFU) on target Vero E6 cells as previously described [40]. In short, Vero E6 cells were seeded at 2 × 10^5^ cells per well in 24-well plates and incubated overnight. Cells were infected with 10-fold serial dilutions of virus stocks and incubated for 1 h. The inoculum was removed and plates were overlaid 1.5% carboxymethylcellulose (Sigma-Aldrich, Taufkirchen, Germany) in MEM culture medium. Plates were incubated for 7 days and then were fixed with 10% formaldehyde and stained with 1% crystal violet (Sigma-Aldrich, Taufkirchen, Germany) in 10% methanol for 20 min to visualize plaques. HCV was titrated as described elsewhere [41]. Infectivity titers were determined using the JFH1 NS5A-specific mouse monoclonal antibody 9E10 (kindly provided by C. Rice, The Rockefeller University, NY) and expressed as the 50% tissue culture infective dose (TCID_50_)/ml.

### 2.7. Gel Electrophoresis and Western Blot Analysis

Denaturing SDS-polyacrylamide gel electrophoresis and Western blotting was performed as described elsewhere [42]. Dilutions of 1:4000 for DENV NS3 monoclonal antibody (GeneTex International Corporation, Hsinchu City, Taiwan), 1:500 for human HIF-1α mouse monoclonal antibody (kindly provided by G. Simos, originally obtained by BD Biosciences, San Jose, CA, USA), 1:1000 for human phosphorylated AKT rabbit monoclonal antibody (Ser^473^, Cell Signaling, Leiden, The Netherlands), 1:100 for GFP rabbit polyclonal antibody (Santa Cruz Biotechnology, Dallas, TX, USA), and 1:6000 for β-actin mouse monoclonal antibody (Merck-Millipore, Burlington, MA, USA), respectively, were used. A dilution of 1:2000 for the secondary anti-mouse and anti-rabbit horseradish peroxidase-conjugated antibodies (Cell Signalling, Leiden, The Netherlands) was used. Imaging quantification was performed by using Quantity I software (Bio-Rad, Hercules, CA, USA).

### 2.8. Luciferase Assays

Firefly luciferase (F-Luc) activity in cell lysate was measured using Luciferase Assay System (Promega Corporation, Madison, WI, USA), as recommended by the manufacturer. *Renilla* luciferase (R-Luc) activity in cell lysates was measured using 12 μM coelenterazine (Promega Corporation, Madison, WI, USA) in assay buffer (50 mM potassium phosphate, pH 7.4, 500 mM NaCl, 1 mM EDTA). Measurements were taken in a GloMax 20/20 single-tube luminometer (Promega Corporation, Madison, WI, USA) for 10 s. Luciferase activities were normalized to the total protein amount determined using the Bradford assay reagent (Bio-Rad, Hercules, CA, USA).

### 2.9. Measurement of Intracellular ATP Levels

ATP was measured using the ViaLight HS BioAssay kit (Lonza, Basel, Switzerland) according to the manufacturer’s protocol in a GloMax 20/20 single-tube luminometer (Promega Corporation, Madison, WI, USA) for 1 s. ATP levels were normalized to total protein amounts.

### 2.10. RNA Quantification by Reverse Transcription—Quantitative PCR (RT-qPCR)

Total cellular RNA was extracted using TRIzol reagent (Thermo Fisher Scientific, Waltham, Massachusetts, USA), according to the manufacturer’s instructions. cDNA synthesis was performed with Moloney murine leukemia virus reverse transcriptase (Promega Corporation, Madison, WI, USA) according to the manufacturer’s protocol and with a mixture of the specific primers DV-A10940 (5’-ACCATTCCATTTTCTGGCGTT-3’) and YWHAZ-R for the DENV positive-strand RNA and the 14-3-3-zeta polypeptide (YWHAZ) mRNA, respectively, DV-S10873 (5’-GAAAGACCAGAGATCCTGCTGTCT-3’) and YWHAZ-R for the DENV negative-strand RNA (3.5 pmol/μl of each primer), or pd(N)6 random hexamer primers (Qiagen, Düsseldorf, Germany) for the cellular transcripts. Real-time quantitative PCR was performed using KAPA SYBR FAST qPCR Master Mix (Sigma-Aldrich, Taufkirchen, Germany) as well as primer pairs specific for the DENV 3’UTR (DV-S10873 and DV-A10940) or the cellular YWHAZ, vascular endothelial growth factor A (VEGFA), glucose transporter 1 (GLUT1), hexokinase 2 (HK2), and lactate dehydrogenase A (LDHA) (for primer sequences see [25]). The YWHAZ housekeeping gene was selected as a normalization control, as it was confirmed that its expression was not affected under low-oxygen conditions [43].

### 2.11. Chemicals

AKT inhibitor VIII (AKTi-1/2) was obtained from Cayman Chemical (Ann Arbor, Michigan, USA) and HIF inhibitor VI (NSC-134754) from Merck Millipore (Burlington, MA, USA). CoCl_2_, DFO, DMOG and reduced L-Glutathione were purchased from Sigma-Aldrich (Taufkirchen, Germany).

### 2.12. Statistical Analysis

In all diagrams, bars represent mean values of at least three independent experiments in triplicate or quadruplicate. Error bars represent standard deviation. Only results subjected to statistical analysis using Student’s *t*-test with *p* ≤ 0.05 were considered statistically significant. Statistical calculations were carried out using Excel Microsoft Office® (Microsoft Corporation, Redmond, WA, USA).

## 3. Results

### 3.1. Low Oxygen Tension Enhances DENV Genome Replication in Cultured Cells

DENV naturally infects hepatocytes, monocytes/macrophages, and endothelial cells that are exposed to an oxygen concentration ranging from 1 to 12% (*v*/*v*) [23]. However, to date, in cell culture, the DENV life cycle has been conventionally studied under atmospheric oxygen conditions (20% O_2_). We have previously shown for the closely related virus HCV (*Flaviviridae* family) that low oxygen favors genome replication in human hepatoma cells through a mechanism that is HIF-independent and directly associated with an increase in anaerobic glycolysis and upregulation of specific oncogenes (i.e., AKT, AP-1) [25]. However, it is unclear whether an analogous mechanism operates for DENV.

**Figure 2:**
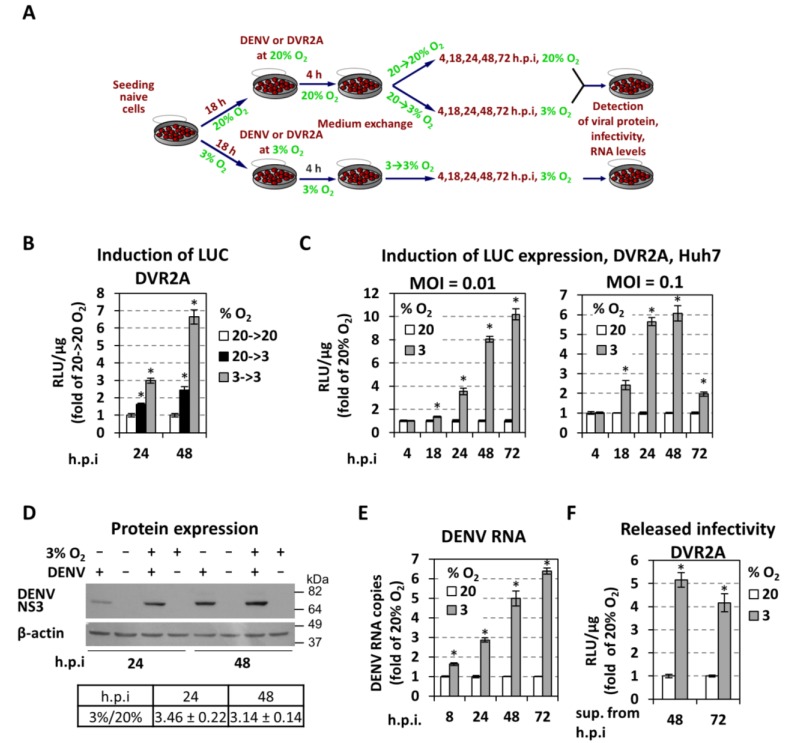
**Low oxygen tension enhances the production of DENV in hepatoma Huh7 cells.** (**A**) Schematic representation of the experimental procedure. Cell culture produced DENV or DVR2a virus stocks were used for infection of naive cells that were seeded at 30% confluence (to avoid pericellular hypoxia) and preincubated for 18 h at 20% or 3% O_2_, respectively. After 4 h cells were washed twice with fresh culture medium, new medium was added and the incubation of cells continued as follows: cells preincubated at 20% O_2_ were further incubated at either 20% (referred to as 20→20% or 20%) or 3% O_2_ (referred to as 20→3%) whereas cells preincubated at 3% O_2_ were further incubated at 3% O_2_ (referred to as 3→3% or 3%). At the indicated time-points, cells were lysed and the expression of virus-related proteins, virus titers, and the amounts of viral RNA were determined. (**B**–**C**) Hypoxic conditions enhance DENV replication. Huh7 cells cultured under specified oxygen conditions were infected with DVR2A at MOI 0.1 (**B**) and MOI 0.1 or 0.01 (**C**), lysed at the indicated time-points and R-luc activity was measured. Values are expressed as RLU/μg of total protein amount and normalized to those obtained with 20→20% (Β) or 20% (C) O_2_ cells (each time-point set to one). (**D**) (Top) Western blot analysis of DENV NS3 protein (top) and β-actin (bottom) of DENV- and non-infected cells, incubated as specified in the top of each lane. Infection was performed with DENV at an MOI of 0.5 and cells were lysed 24 or 48 h p.i. β-actin served as a loading control. Condition of 20% O_2_ is indicated as “−“ and 3% O_2_ as “+“. Numbers on the right refer to the positions of molecular mass marker proteins. A representative experiment is shown. (Bottom) Image quantification of NS3 signals (mean values from 3 independent repetitions), normalized to β-actin and to the values obtained with cells cultured under 20% O_2_. (**E**) Viral RNA copies in cells infected with DENV at MOI = 0.01 were determined by RT-qPCR. YWHAZ mRNA levels were used for normalization. Values obtained with 20% O_2_ cells were set to one for each time-point. (**F**) Virus amounts released from Huh7 cells previously infected with DVR2A (MOI = 0.1) at the indicated oxygen conditions. Supernatants were collected at 48 and 72 h p.i. and used to infect naive Huh7 cells (infected and incubated at 20% O_2_), 72 h post-infection the cells were lysed and luciferase activity was measured and normalized to total protein amount. Values obtained with 20% O_2_ cells were set to one. In all panels, bars represent mean values from at least three independent experiments in triplicate. Error bars indicate standard deviations. * *p <* 0.001 vs. 20% O_2_ cells, for 8–72 h p.i (Student’s *t*-test).

To investigate the effect of low oxygen on DENV RNA replication in Huh7 hepatoma cells, we compared viral replication in cells at normoxic conditions or under hypoxia mimicking the liver microenvironment (3% *v*/*v* O_2_) using the schedule depicted in Figure 2A. Cells were infected with a highly replication competent derivative of the DENV2 strain 16681 encoding the *Renilla* luciferase (R-Luc) reporter (DVR2A; Figure 1), and virus replication was evaluated as depicted in Figure 2A by using R-Luc assay. A comparison of replication levels at 24 and 48 h post-infection (h p.i) revealed a remarkable increase when cells were kept under hypoxic conditions already prior to infection (3→3% O_2_ condition) (Figure 2B).

For a detailed kinetic analysis of DVR2A replication, we compared hypoxic and normoxic conditions during a time course of 72 h p.i and using two different multiplicities of infection (MOIs - 0.01 and 0.1). DENV replication was increased 6- to 10-fold under hypoxic conditions depending on the MOI (Figure 2C; Figure S1A). Specifically, higher increase of viral replication with greater duration, but delayed kinetics, was observed with the lower MOI, possibly reflecting a less drastic exhaustion of host cell factors. Moreover, the enhancement was affected by cell confluency (Figure S1B), which was most likely due to the development of pericellular hypoxia [44] at high cell densities (Figure S1D). Accordingly, enhanced accumulation of NS3 protein (~3-fold; Figure 2D) and viral RNA (up to ~6-fold; Figure 2E) was detected in lysates of DENV-infected Huh7 cells cultured under hypoxic conditions. Moreover, a significant increase of virus released from cells cultured at 3% O_2_ (4- to 5- fold for MOI = 0.1; Figure 2F) was detected. Overall, our results show that low oxygen provides an advantage to DENV replication in Huh7 cells. 

**Figure 3:**
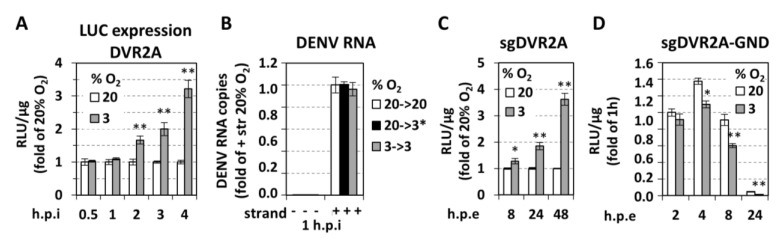
**Low oxygen tension selectively enhances DENV RNA replication.** (**A**) Huh7 cells preincubated at 20% or 3% O_2_ for 18 h were inoculated with DVR2A (MOI = 1) and lysed at the specified time-points post inoculation. Luciferase activity is expressed as RLU/μg of total protein amount and values obtained with 20% O_2_ cells were set for each time point to one. (**B**) Hypoxia does not influence viral entry. RT-qPCR analysis of intracellular DENV positive (+) strand RNA copies from Huh7 cells that were inoculated with DENV at MOI = 1 and incubated for 1 h as specified. 20→3*% O_2_ refers to cells that were preincubated at 20% O_2_ and transferred immediately after virus inoculation from 20% to 3% O_2_. Negative (−) strand RNA was quantified in order to indicate the absence of viral replication at 1 h post-inoculation. Values are expressed relative to the positive-strand RNA obtained at 20→20% O_2_. (**C**,**D**) Hypoxia increases viral RNA replication but not translation. Huh7 cells preincubated at 20% or 3% O_2_ for 18 h, were electroporated (5 μg RNA/4 × 10^6^ cells) with subgenomic sgDVR2A (sgDV, **C**) or its replication defective variant, sgDVR2A-GND (GND, **D**), and further incubated at the preincubation conditions. Cells were lysed at the indicated time-points and luciferase activity is expressed as RLU/μg of total protein amount. Luciferase levels measured one hour post-electroporation (h.p.e.) were used for normalization for each construct and oxygen condition. For sgDV (**C**), values obtained at 3% O_2_ are expressed as fold of the respective ones at 20% O_2_. For GND (D), values obtained under 20% O_2_ at 2 h, were set to 1. In all panels, bars represent mean values from at least three independent experiments in triplicate. Error bars indicate standard deviations. * *p* < 0.01, ** *p* < 0.001 vs. 20% O_2_ cells (Student’s *t*-test).

With the aim to determine the exact step of the viral replication cycle affected by oxygen tension, we determined the kinetics of replication enhancement after inoculating cells for 0.5 up to 4 h with DVR2A at an MOI of 1. As shown in Figure 3A and Figure S1E, an increase of luciferase activity in cells cultured at 3% O_2_ appeared already at 2 h post-inoculation arguing that either virus entry, RNA translation, or an early step of the RNA replication cycle is affected. To address virus entry, we quantified DENV positive-strand RNA level 1 h after inoculation, reflecting viral RNA introduced into cells prior to the onset of RNA replication as deduced from the absence of negative-strand RNA at this time point (Figure 3B). We observed that viral positive-strand RNA levels were comparable between cells cultured under various oxygen conditions, suggesting that virus entry is not affected by low oxygen in our system. To support this assumption, we electroporated the bicistronic reporter replicon sgDVR2A (Figure 1) into Huh7 cells incubated under normoxic or hypoxic conditions. A ~2 and 3.5-fold increase of RNA replication in cells kept under hypoxic relative to normoxic, conditions, respectively, was found (Figure 3C; Figure S1F). While these results confirm that hypoxia facilitates DENV RNA translation or replication, but not virus entry, the lower replication enhancement observed with transfected cells, as compared to infected cells (Figure 2C), might be due to the different kinetics of viral RNA replication resulting from the different delivery of viral RNA into cells.

Next, we investigated whether low oxygen tension affects DENV RNA translation. For this, a non-replicative bicistronic DENV reporter RNA (sgDVR2A-GND) (Figure 1) was electroporated into Huh7 cells that were kept under normoxic or hypoxic conditions for 2–24 h. Interestingly, a reduction of luciferase activity was detectable with cells maintained at 3% O_2_ (Figure 3D), arguing that either RNA stability or translation was reduced under hypoxic conditions. However, as DENV RNA translation is cap-dependent and the rate of cellular protein synthesis is reduced under hypoxia [45] as shown previously [25], reduced RNA translation of viral RNA under hypoxic conditions is the more likely explanation. We note that the positive effect of low oxygen tension on DENV RNA replication was confirmed in various other cell systems: an immortalized human hepatocyte (IHH) cell line [46,47] (Figure S2A), a monocytic cell line (THP1; Figure S2C), with monocytes being the primary target cells of DENV, and in Vero E6 cells (Figure S2E).

### 3.2. Low Oxygen-Mediated Enhancement of DENV Replication is Linked to Hypoxia-Induced Reprogramming of Cellular Energetics

**Figure 4:**
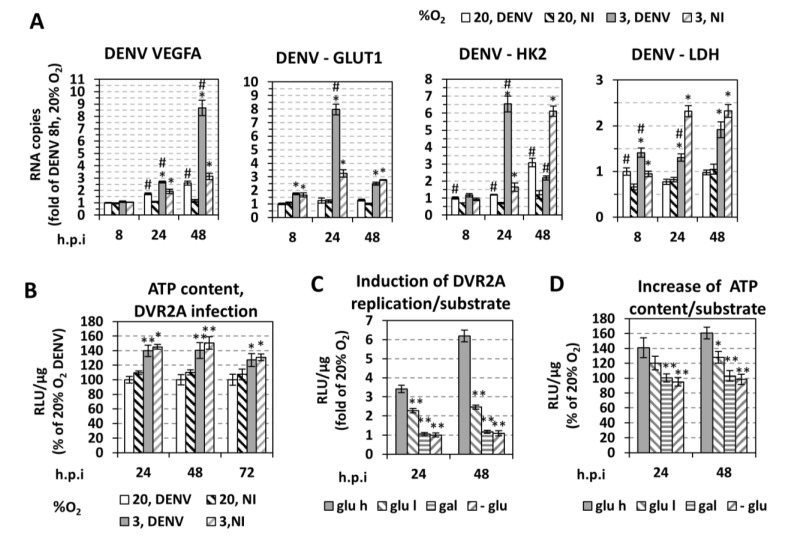
(**A**,**B**) **Hypoxia induces transcriptomic and metabolic reprogramming in DENV-infected cells.** (**A**) RT-qPCR analysis of VEGFA, GLUT1, HK2, and LDH mRNA in DENV-infected (MOI = 0.5) or non-infected Huh7 cells incubated at the specified oxygen conditions and lysed at the indicated time points p.i. YWHAZ mRNA levels were used for normalization**.** Values obtained with infected cells at 20% O_2_ and lysed 8 h.p.i. were set to one. Mean values from three independent experiments are presented. * *p* < 0.001 vs. 20% O_2_ cells, ^#^ *p* < 0.01 vs. NI cells (Student’s *t*-test). (**B**) Intracellular ATP levels of Huh7 DVR2A-infected (MOI 0.1) and non-infected cells, incubated at the specified oxygen conditions. Cells were lysed at the indicated time points p.i. and intracellular ATP levels were expressed as RLU/μg of total protein amount. Values from cells cultured at 20% O_2_ at each time-point were set to 100. * *p* < 0.01, ** *p* < 0.001 vs. 20% O_2_ cells. (**C,D**) **Association between glucose metabolism and low oxygen-mediated DENV replication enhancement.** Fold difference between 3% and 20% O_2_ of viral replication-derived luciferase activity (**C**) and intracellular ATP levels (**D**) in DVR2A-infected cells (MOI 0.01) incubated in culture media that differ in glucose concentration: high glucose (25 mM, glu h), low glucose (5.56 mM, glu l), galactose (10 mM, gal) instead of glucose or no glucose (-glu). Cells were lysed at the indicated time points p.i. In all panels, bars represent mean values from at least three independent experiments in triplicate. Error bars indicate standard deviations. * *p* < 0.001, ** *p* < 0.001 vs. glu h cells at 3% O_2_.

To examine the reprogramming of cellular bioenergetics by low oxygen [24,48], we compared non-infected (NI) and DENV-infected cells with respect to changes in the expression of genes related to hypoxia and energy content (ATP levels). By using RT-qPCR analysis of Huh7 cells cultured up to 48 h at 3% O_2_ we observed an upregulation of selected hypoxia-related genes (Figure 4A), i.e., VEGFA, which is the direct transcriptional target of HIFs, as well as genes involved in glucose transport (GLUT1) and anaerobic glycolysis (HK2, LDH-A). These results confirm a metabolic shift towards increased anaerobic glycolysis under hypoxic conditions. This assumption was supported by observed elevations of intracellular ATP levels in DVR2A-infected Huh7 cells (Figure 4B), IHHs (Figure S2B), THP-1 (Figure S2D), and Vero E6 cells (Figure S2F). No significant difference in the ATP content was observed between infected and control cells (Figure 4B). Notably, ATP level was cell confluence-dependent, most likely due to pericellular hypoxia at high cell densities [44] (see Figure S1C). In conclusion, these data argue for a hypoxia-mediated bioenergetic reprogramming.

To test the relationship between cell energetics and DENV replication, we modulated ATP production of cells by feeding them with high or low glucose, galactose, or no glucose containing medium, followed by incubation of cells at different O_2_ concentrations for 24–48 h, when the peak of hypoxia-mediated enhancement of viral replication is observed (Figure 2). Galactose was used in order to shift the energetic metabolism towards oxidative phosphorylation. Glucose reduction or substitution reduced both the intracellular ATP content and DENV replication in hypoxic cells in a glucose concentration-dependent manner (Figure 4C,D). Thus, DENV replication enhancement under hypoxia correlates with an increase in anaerobic glycolysis, concomitant with increased ATP production.

### 3.3. Low Oxygen-Related Enhancement of DENV RNA Replication is Mediated by HIF-α

**Figure 5:**
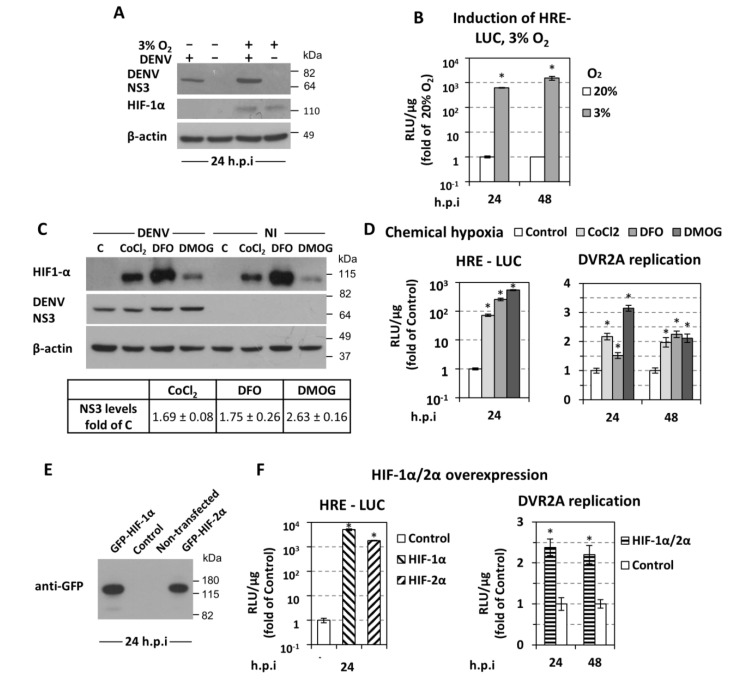
**HIF upregulation enhances DENV replication.** (**A**) Western blot analysis of DENV NS3 (top), HIF-1α (middle), and β-actin (bottom) of DENV-infected (MOI 0.5) or non-infected cells, incubated as specified on top of each lane and lysed at 24 h p.i. β-actin served as a loading control. A representative experiment of 3 independent repetitions is shown. (**B**) Activation of HRE (hypoxia response element) by low oxygen. Huh7 cells were transfected with the 9×HRE-Luc construct (0.4 μg/4 × 10^4^ cells), 4 h post-transfection inoculated with DENV (MOI 0.5) for 4 h and further incubated at 20% or transferred to 3% O_2_ for 24 or 48 h p.i. HRE-dependent F-Luc activity was measured and expressed as RLU/μg of total protein amount. Values obtained from cells incubated at 20% O_2_ were set to one each time. * *p* < 0.001 vs. 20% O_2_ cells (Student’s *t*-test). (**C,D**) Chemically-induced hypoxia stabilizes HIF-1α and enhances DENV replication. (**C**) (Top) Western blot analysis of HIF-1α (top) DENV NS3 (middle) and β-actin (bottom) of Huh7 cells inoculated with DENV (MOI 0.5) for 4 h, and subsequently treated, for 24 h, with CoCl_2_ (75 μM), DFO (37.5 μM), or DMOG (62.5 μM), as specified on top of each lane. β-actin served as loading control. A representative experiment is shown. C: control non-treated cells. (Bottom) Quantification of NS3 signals from 3 independent experiments, normalized to the β-actin loading control was performed and mean values are expressed relative to that obtained from control cells. (**D**) Luciferase activity obtained with Huh7 cells transfected with the 9×HRE-Luc construct (left) or infected with DVR2A (MOI = 0.01, right) were treated with CoCl_2_ (75 μM), DFO (37.5 μM), or DMOG (62.5 μM) at 6 h post-transfection or 4 h post virus inoculation, respectively. Cells were lysed at the indicated time-points. Mean values are expressed relative to the reporter activity derived from control-non treated cells. (**E**,**F**) Overexpression of HIF-1α and HIF-2α enhances DENV replication. Huh7 cells were either co-transfected with the 9×HRE-Luc construct and a plasmid that expresses GFP-HIF-1α, GFP-HIF-2α, or empty vector (control), or first transfected with both HIF-expressing plasmids and 18 h later infected with DVR2A (MOI 0.1). (**E**) Western blot analysis of GFP-HIF-1α and GFP-HIF-2α using an anti-GFP antibody. (**F**) HRE-dependent F-Luc (left) and DVR2A-derived R-Luc (right) were measured and expressed as RLU/μg of total protein amount. Values of control cells were set to one for each time point. In all panels, bars represent mean values from at least three independent experiments in triplicate. Error bars indicate standard deviations. *p < 0.001 vs. control cells.

Hypoxia-inducible factors (HIFs) are important transcription factors for cell adaptation to hypoxia [24]. They are stabilized and activated under conditions below 5% O_2_ due to reduced hydroxylation by prolyl hydroxylation domain enzymes (PHD or EGLN) [49]. The oxygen-regulated HIF-α subunits and the ubiquitous HIF-β form a complex that interacts with HRE-containing promoters. Moreover, HIF-1α activation under normoxia is a general phenomenon in bacterial, protozoan, and viral infections [31]. Among *Flaviviridae* viruses, HCV is known to induce HIF-1α stabilization at late time points post-infection [25,50,51,52]. Therefore, we examined whether the enhancement of DENV replication observed under hypoxic conditions was related to HIF activation by performing the following experiments:

First, we confirmed that HIF-1α was stabilized in hypoxic Huh7 cells infected with DENV (Figure 5A). We also confirmed an ~1000-fold activation of a minimalized HIF-dependent promoter (HRE) in hypoxic DENV-infected cells (Figure 5B).

Second, we pharmacologically induced HIF stability and/or activity [53] by treating Huh7 cells with non-toxic concentrations (Figure S3A) of CoCl_2_, desferrioxamine (DFO), or dimethyloxallyl glycine (DMOG), all increasing HIF abundance in the cells (Figure 5C) and HRE activity (Figure 5D, left). Under those conditions, we observed an up to 3-fold increase in DENV replication as determined by Western blot analysis of NS3 (Figure 5C), and by measuring DENV RNA replication via luciferase activity (Figure 5D right). These results suggest that hypoxia-related enhancement of DENV replication is dependent on HIF activation.

Third, we overexpressed the two best studied HIF-α isoforms, HIF-1α and -2α [54] (Figure 5E), each one leading to an upregulation of HRE (Figure 5F, left), comparable to the one observed under hypoxia, and increasing DVR2A replication (Figure 5F, right).

**Figure 6:**
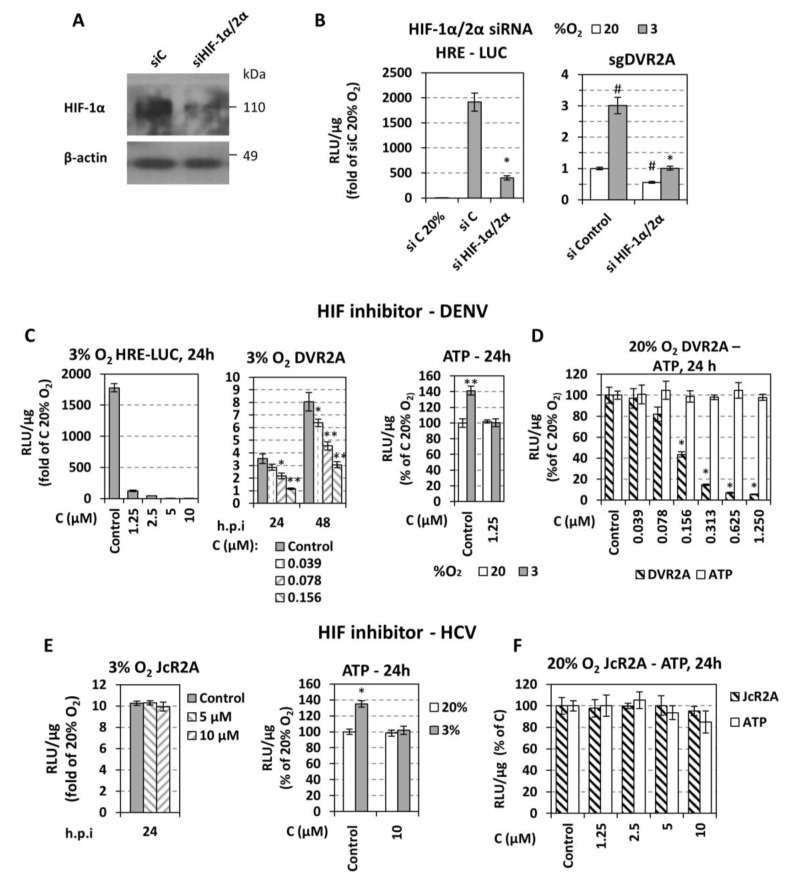
**HIF downregulation impairs the replication of DENV but not HCV.** (**A**,**B**) Huh7 cells were co-transfected with a mixture of siRNAs targeting HIF-1α and HIF-2α (20 nM each) or a control siRNA (40 nM) and the 9×HRE-Luc construct, or the mixture of siHIF-1α/2α (or the control siRNA) and subgenomic sgDVR2A (sgDV). (**A**) Western blot analysis of endogenous HIF-1α protein (top) and β-actin (bottom) of cells incubated at 3% O_2_ for 24 h. β-actin served as loading control. (**B**) HRE-dependent F-Luc (left) and sgDVR2A-dependent R-Luc (right) activities from cells incubated at 20% or 3% O_2_ for 24 h were measured and expressed as RLU/μg of total protein amount. Values were normalized to the reporter activity detected in cells cultured at 20% O_2_. * *p* < 0.001 vs. si control cells at 3% O_2_, # *p* < 0.001 vs. si control cells at 20% O_2_ (Student’s *t*-test). (**C**,**D**) Huh7 cells pre-incubated at 20% or 3% O_2_ were transfected with the 9×HRE-Luc construct or inoculated with DVR2A (MOI 0.01) for 4 h. Subsequently, cells were treated with serial dilutions of the HIF-1 inhibitor NSC-134754 and further incubated at the pre-incubation condition. Reporter activity was measured and expressed as RLU/μg of total protein amount. Values are expressed as ratio of the respective ones measured at 3% versus 20% O_2_ (C left, middle), or as percentage of the ones from control-treated cells at 20% O_2_ (C right, D). For panel C: * *p* < 0.01, ** *p* < 0.001 vs. control cells. For panel D: * *p* < 0.001 vs. control cells. (**E**,**F**) Huh7.5 cells were infected with the HCV reporter virus JcR2a (MOI = 1), treated with the HIF-1 inhibitor NSC-134754 and cultured as described for DENV (**C**,**D**). In all panels, bars represent mean values from at least three independent experiments in triplicate. Error bars indicate standard deviations. * *p* < 0.001 vs. control cells.

Finally, we evaluated the role of HIFs in the hypoxia-mediated enhancement of DENV replication by silencing HIF-1α and 2α expression or inhibiting HIF-1 with the small-molecule inhibitor NSC-134754. A mixture of two siRNAs (siHIF1α/2α) reduced efficiently HIF-1α levels at 3% O_2_ in cells transfected with sgDVR2A (Figure 6A) and down-regulated ~5-fold HRE activity (Figure 6B left). Importantly, HIF knock-down reduced the enhancement of DENV RNA replication in hypoxic cells (Figure 6B right). Consistently, pharmacological HIF-1 inhibition, confirmed by HRE downregulation (Figure 6C left), dose-dependently reduced DENV replication enhancement (Figure 6C middle) without affecting cell viability (Figure 6D). Moreover, this HIF-1 inhibition decreased ATP levels that were otherwise increased in hypoxic mock-treated cells (Figure 6C right). Consistent with our earlier report, pharmacological HIF inhibition did not affect HCV replication in hypoxic cells (Figure 6E left), even though ATP level was reduced under those conditions (Figure 6E right). These results argue against a pleiotropic effect of HIF depletion/inactivation and are in favor of a specific enhancement of DENV RNA replication.

Interestingly, HIF-1 inhibitor at concentrations ≥0.1 μΜ had a detrimental effect on DVR2A replication even at 20% O_2_ (Figure 6D), which is consistent with the reduction of viral replication observed upon HIF silencing under the same conditions (Figure 6B, right). On the contrary, HCV replication was HIF-independent (Figure 6F).

### 3.4. Role of AKT for Enhancement of DENV Replication Under Hypoxic Conditions

**Figure 7:**
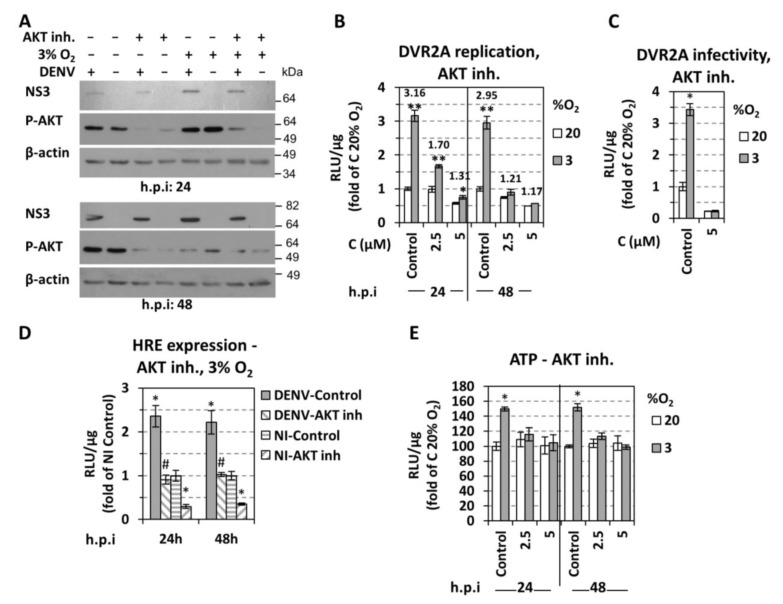
**Effect of AKT inhibition on oxygen-regulated increase of DENV replication.** (**A**–**E**) Huh7 cells, preincubated at 3% or 20% O_2_ for 18 h, were infected with DENV (MOI 0.5) or DVR2A (MOI 0.1), or first transfected with the 9×HRE-Luc construct and 4 h later infected with DENV (MOI 0.5). Non-infected cells were used as control. After virus inoculation, the AKT inhibitor VIII was added at 5 μΜ, unless otherwise specified, and cells were further incubated at the pre-incubation conditions. (**A**) Western blot analysis of DENV NS3 (top), p-AKT (middle), and β-actin (bottom). The latter served as loading control. A representative experiment of 3 independent repetitions is shown. (**B**) DVR2A-derived R-Luc activity (RLU/μg of total protein) from infected cells. Values obtained from control-DMSO treated cells incubated at 20% O_2_ were set to one for each time point. Fold difference of values measured at 3% O_2_ versus the corresponding ones at 20% O_2_ are depicted on the top of the bars. * *p* < 0.01, ** *p* < 0.001 vs. 20% O_2_ cells (Student’s *t*-test). (**C**) Release of infectivity of Huh7 cells, previously infected with DVR2A at MOI 0.1 at the indicated oxygen conditions and treated with AKT inhibitor VIII. Supernatants from these cells were collected at 48 h p.i. and used to infect naïve Huh7 cells (infected and incubated at 20% O_2_), 72 h post-infection the cells were lysed and the luciferase activity was measured and expressed as RLU/μg of total protein amount. Values obtained using supernatants of control-DMSO treated cells incubated at 20% O_2_ were set to one. * *p* < 0.001 vs. 20% O_2_ cells. (**D**) HRE-derived F-Luc activity (RLU/μg of total protein) from infected or non-infected cells at 3% O_2_. Values obtained from control-DMSO treated non-infected cells were set (each time) to one. * *p* < 0.001 vs. control NI cells, # *p* < 0.001 vs. control DENV-infected cells. (**E**) Intracellular ATP levels (RLU/μg of total protein) from infected cells. Values obtained from control-DMSO treated cells incubated at 20% O_2_ were set (each time) to 100. * *p* < 0.001 vs. 20% O_2_ cells.

In addition to HIF, the serine/threonine kinase AKT is another major regulator of bioenergetic reprogramming towards anaerobic glycolysis. AKT has been shown to be directly activated by hypoxia in a HIF-independent, but prolyl hydroxylation (hydroxylase EglN1) dependent manner [55,56]. Moreover, AKT is one of the primary upstream regulators of HIF-1 [57,58,59]. Therefore, we hypothesized that AKT might be involved in the modulation of cell hypoxic and energetic status favoring DENV RNA replication. To address this assumption, we used the AKT selective inhibitor VIII (AKTi-1/2) in DENV infected cells cultured at normoxic or hypoxic conditions. Consistent with previous studies [56,60,61], we observed a significant increase of AKT phosphorylation in hypoxic Huh7 cells that was higher (~1.2-fold for DENV-infected, ~3.5-fold for non-infected) as compared to cells kept under normoxic conditions at 24 h p.i. (Figure 7A, upper panel). The AKT inhibitor VIII reduced AKT phosphorylation as expected and caused a gradual reduction of DENV replication enhancement in hypoxic cells as determined by Western blot and luciferase assay (Figure 7A–C). AKT inhibition also lowered HIF activation as determined by HRE-luc promoter assay (Figure 7D), and reversed the ATP increase observed in hypoxic cells (Figure 7E)_._ These results suggest that AKT contributes to the enhancement of DENV replication under low oxygen. However, this enhancement appears to be independent from the upstream effector of AKT, i.e., PI3K, as it was not negatively affected by the pan-PI3K specific inhibitor LY294002 (Cayman, Ann Arbor, MI, USA) (Figure S4).

### 3.5. Effect of DENV on Hypoxic and Metabolic Reprogramming

**Figure 8:**
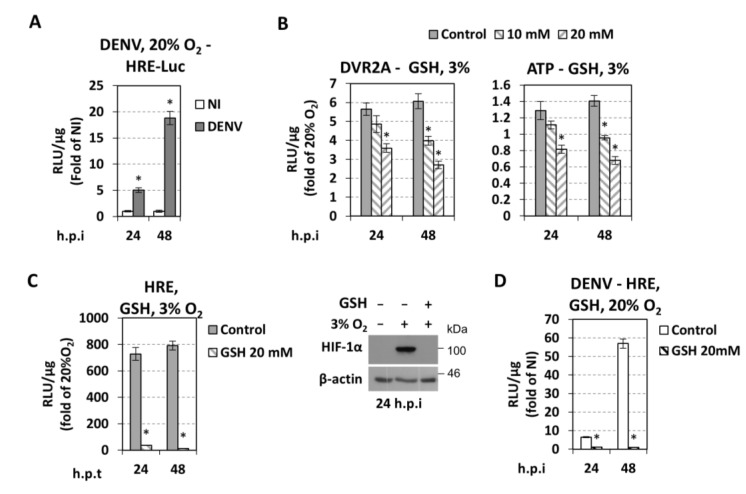
**DENV induces hypoxic reprogramming—Role of redox homeostasis.** (**A**) DENV upregulates HRE at 20% O_2_. Huh7 cells were transfected with the 9×HRE-Luc construct (0.4 μg/4 × 10^4^ cells) and 4 h post-transfection infected, or not, with DENV (MOI 0.5). Cells were further incubated at 20% O_2_. HRE-dependent F-Luc activity was measured and expressed as RLU/μg of total protein amount. Values obtained from non-infected cells were set to one each time. * *p* < 0.001 vs. NI cells (Student’s *t*-test). (**B**) Glutathione reduces hypoxia-induced DENV replication enhancement. Huh7 cells, preincubated at 3% or 20% O_2_ for 18 h, were infected with DVR2A (MOI 0.01). After virus inoculation, reduced glutathione (GSH) was added at the specified concentrations and cells were further incubated at the pre-incubation conditions. DVR2A-derived R-Luc activity and intracellular ATP levels were measured and expressed as RLU/μg of total protein. Values are expressed as ratio of the ones measured at 3% versus 20% O_2_. (**C**) Huh7 cells, were transfected with the 9×HRE-Luc construct treated with glutathione (20 mM) and incubated at 3% or 20% O_2_. (**C**, **left**) HRE-dependent F-Luc activity was measured and expressed as RLU/μg of total protein amount. Values are expressed as ratio of the ones measured at 3% versus 20% O_2_. (**C**, **right**) Western blot analysis of HIF-1α (top) and β-actin (bottom). The latter served as loading control. A representative experiment of 3 independent repetitions is shown. (**D**) Glutathione reduces DENV-mediated HRE activation at 20% O_2_. Huh7 cells were transfected with 9×HRE-Luc construct and 4 h post-transfection were infected, or not, with DENV (MOI 0.5). Subsequently, cells were treated with glutathione (20 mM) and incubated at 20% O_2_. HRE-dependent F-Luc activity was measured and expressed as RLU/μg of total protein amount. Values from DENV-infected cells are presented as fold of the ones derived from non-infected cells. * *p* < 0.001 vs. control cells.

The detrimental effect of HIF inhibition or silencing on DENV replication in normoxic cells prompted us to investigate if DENV infection triggers a hypoxic reprogramming. For this, we first determined the effect of DENV infection on HIF activation under normoxia by transfecting cells with the 9×HRE-Luc reporter construct and 4 h later, infecting them with DENV. A 5- and 20-fold upregulation of HRE at 24 and 48 h p.i. was detected, respectively (Figure 8A). Consistently, under hypoxia, an additional HRE activation (Figure 7D) and HIF-1α protein level increase was observed (Figure 5A,C, Figure S3B) after DENV infection. Interestingly, this virus-mediated HRE upregulation appears to be AKT-independent, as the ratio of HRE levels in DENV-infected versus non-infected cells was not affected by the AKT inhibitor VIII (Figure 7D). However, consistent with previous studies [16,17], DENV infection induces phosphorylation of AKT at 24 h p.i. under normoxia by ~1.7-fold, as compared to non-infected cells (Figure 7A, upper panel). This induction contributes to viral replication, as shown by the ~2-fold reduction in replication after treatment with AKT inhibitor VIII (Figure 7B). Hypoxic (VEGFA) and anaerobic glycolysis markers (GLUT1, HK2, LDH-A) were also induced after DENV infection at early time points (up to 4-fold at 24 h, Figure 4A). These data suggest that DENV induces a hypoxic response and reprograms host cell energy metabolism.

### 3.6. Cellular Redox Homeostasis Under Hypoxic Reprogramming and DENV Infection

Hypoxia has been reported to induce reactive oxygen species (ROS) production due to effects on the mitochondrial electron transport chain, NADPH oxidases or xanthine oxidase [62,63]. Although it is not clear whether ROS release under hypoxia has a direct role on HIF-1α stabilization, there is increasing evidence that nitric oxide, specific microRNAs, ERK, and AKT pathways are involved in ROS mediated regulation of HIF-1α [62,64]. Moreover, alterations in redox homeostasis upon DENV infection have been previously recognized under normoxic conditions in cell culture, in mice, and in patients [65,66,67,68,69,70]. A major mechanism used by cells to reduce the levels of ROS involves the production of glutathione and its utilization in the reaction H_2_O_2_ + 2GSH → GSSG + 2H_2_O, where GSH represents reduced glutathione [71]. In addition, glutathione was reported to have an inhibitory effect on DENV production in hepatoma cells [72].

Therefore, we investigated whether the alteration of DENV replication by hypoxia is mediated by ROS production. First, we confirmed an elevation of ROS levels in cells cultured under hypoxic conditions (FACS analysis, Figure S5A). Then we evaluated a possible role of glutathione for DENV replication under hypoxic conditions. Huh7 cells were inoculated with DVR2A for 4 h and then treated or not with glutathione using concentrations that do not impact cell growth (Figure S5C), and have antioxidative activity (Figure S5D) as previously reported [73,74]. Glutathione significantly reduced DENV replication enhancement in hypoxic cells (Figure 8B, left and Figure S5C). Glutathione also reduced ATP levels in hypoxic cells (Figure 8B, right) and had a negative effect on the activation of HRE and HIF stabilization (Figure 8C), suggesting that hypoxia-induced ROS might enhance viral replication, at least in part, by HIF. As control experiments, we showed that H_2_O_2_-induced ROS has a positive impact on DVR2A replication (i.e., negative and positive strand RNA amounts) (Figure S5E) and on HIF stabilization (Figure S5F), after treatment of cells with H_2_O_2_. On the other hand, DENV stimulated ROS production under normoxic conditions as determined by FACS analysis (Figure S5B) and activated the HRE promoter, which was nullified by glutathione (Figure 8D). These results suggest that the cellular redox homeostasis is responsible for DENV-mediated hypoxic reprogramming.

## 4. Discussion

Recent studies have shown that oxygen tension exerts a profound effect on the replication of several viruses [28]. In general, hypoxia restricts the replication of viruses that naturally infect tissues exposed to ambient oxygen (Influenza virus, Adenovirus) [75,76] and enhances the replication of viruses that naturally target tissues exposed to low oxygen (Vesicular Stomatitis virus, Herpes viruses, Human Immunodeficiency virus, Parvovirus B19) [77,78,79,80]. We have previously shown that low oxygen tensions, simulating the physiological status of the liver (3–12% O_2_), favor the replication of the *Flaviviridae* virus HCV [25] in a HIF-independent and anaerobic glycolysis dependent manner.

To date, DENV in vitro infection has been studied under normoxic conditions, although the microenvironment of target tissues, including liver and monocytes that traffic to lymph nodes and the spleen, is hypoxic [23]. There is only one report, based on monocytes, showing hypoxia-enhanced DENV infection through an unknown post-uptake mechanism [29]. Here, we investigated DENV replication under hypoxic conditions and made several observations that will be discussed in the following paragraphs.

### 4.1. Low Oxygen Tension Enhances DENV Replication in Cultured Hepatocytes, Monocytes and Epithelial cells

We observed hypoxia-induced DENV replication in human hepatoma cells (Figure 2) as well as in a human hepatocyte cell line [46] retaining important features of normal hepatocytes, including the secretion of liver-specific plasma proteins (albumin, fibrinogen, apoB) [47]. Hypoxia also enhanced DENV replication in monocytes (THP1, [81]), the primary target cells of DENV and in epithelial cells (Vero E6). Enhancement was most pronounced by preincubation of cells at 3% O_2_ prior to infection arguing that preexisting hypoxia-induced cellular factor(s) promote DENV replication. We found that hypoxia increased an early step of viral RNA replication, whereas virus entry, RNA translation, virion assembly, and release were not affected (Figure 2 and Figure 3).

### 4.2. Low Oxygen-Mediated Increase in DENV Replication in Cultured Cells is Directly Linked to Cellular Energetic Changes

Hypoxia is known to be associated with an adaptive cell metabolic reprogramming, including a shift in glucose metabolism from oxidative phosphorylation to anaerobic glycolysis and lactic acid production [24,48], which had also been shown in hepatocytes [25,82] and monocytes [26,27]. For highly proliferating cells, the advantage of such a metabolic switch is to combine energy (ATP) from enhanced glycolysis with the production of nutrients/intermediates for cell growth and division [83]. HIF and AKT are two major regulators of bioenergetic reprogramming towards anaerobic glycolysis, directly activated by hypoxia in a prolyl hydroxylation-dependent manner [24]. Consistent with the above and an earlier report [84], we found that hypoxia-induced glycolysis increased both intracellular ATP levels (Figure 4D) and DENV replication (Figure 4C). However, oxidative phosphorylation, although active at 3% O_2_, had no role in the observed gain of energy as determined by pharmacological inhibition of mitochondrial ATP synthase in infected cells (data not shown). Conversely, DENV infection increased glucose metabolism rate at early time-points (8, 24 h p.i.), as determined by the upregulation of genes involved in glucose uptake (GLUT1) and anaerobic glycolysis (HK2, LDH-A). In the case of GLUT1, virus-mediated induction was significant only under hypoxia, consistent with previous results on GLUT1 [84]. We note that at late time points post-infection (48 h), with high virus titer, this upregulation was no longer detectable (GLUT1) or even reverted (HK2, LDH), which is expected as DENV, after 24 h of infection, is known to reduce cell viability and promote apoptosis [15,16].

### 4.3. Low Oxygen-Mediated Increase of DENV Replication Depends on HIF-α Activation

HIFs are fundamental control transcription factors of the cellular metabolic state under low oxygen [24]. Low oxygen-dependent enhancement of DENV replication correlated directly with HIF-1α/2α chemical induction and overexpression (Figure 5), while silencing and pharmacological inhibition blunted the enhancement (Figure 6). Interestingly, DENV replication was shown to be HIF-dependent even at 20% O_2_. This is possibly due to a positive feedback between DENV and HIF, because viral infection increases the expression of the HIF-target gene VEGFA (Figure 4) and upregulates HRE activation (Figure 8). Moreover, under hypoxic conditions, DENV caused an additional induction in HIF-1α levels and HRE activity (Figure 7). This DENV-mediated HIF activation appears to be independent of AKT (Figure 7D). Overall our data support a bidirectional relationship between DENV and HIF, which is established early during infection and favors viral replication efficiency. Taken together, these results suggest that DENV induces cellular hypoxic response and reprogramming of energy metabolism, and based on the abovementioned favorable role of hypoxia, this possibly occurs in order to support efficient viral replication. Consistently, a number of viruses also exploit HIF stabilization/activation to promote their replication, through mechanisms that include PHD degradation, inhibition of von Hippel–Lindau protein (VHL) and HIF-α binding (through direct association with HIF-α, or other mechanisms), activation of PI3K/AKT or MAPK pathways and ROS production [28]. Such mechanisms could be envisaged for the association between DENV and HIF.

### 4.4. Different Roles of Hypoxia-Related Factors in HCV and DENV Replication

We have reported earlier that hypoxia also increases HCV RNA replication, but in a HIF-independent manner [25] (Figure 6E). Thus, enhancement of RNA replication by hypoxia appears to be conserved within *Flaviviridae* viruses. However, there is a distinct role of HIF on DENV and HCV enhancement, which is consistent with the different kinetics of HIF induction by the two viruses. Specifically, HIF has been activated already 24 h post DENV infection (Figure 8A), but not earlier than 72 h post HCV infection [25,50,51,52].

Another difference between DENV and HCV relates to AKT. We found that AKT phosphorylation was associated with the upregulation of DENV replication under hypoxic conditions. Moreover, AKT was shown to control, at least in part, HRE activity and cell bioenergetics (intracellular ATP) under those conditions (Figure 7D,E). This is again different to HCV for which we suggested a direct, HIF-independent, role of AKT in regulating virus replication under hypoxic conditions [25]. Interestingly, the upstream effector of AKT, PI3K, neither accounts for the role of AKT in DENV replication enhancement, nor for the increase of cellular ATP content occurring under low oxygen tension (Figure S4). These results are in agreement with a previous report showing no alteration in DENV replication upon PI3K inhibition [16].

### 4.5. Role of Cellular Redox Homeostasis in DENV Replication

We found that ROS induction by hypoxia-mediated DENV replication increase, as well as DENV-induced ROS under normoxic conditions contributed to HIF activation (Figure 8). Moreover, the negative effect of glutathione on the DENV replication enhancement under hypoxic condition correlated with a downregulation of HIF activation/stabilization. Based on the previously reported link between ROS and HIF-1α stabilization [62,64], which was confirmed in our studies (Figure S5), we propose that the positive role of hypoxia-induced ROS in viral replication might be mediated, at least in part, by HIF. Thus, we suggest that DENV induces ROS as a means of HIF stabilization, to create an environment simulating tissue hypoxia, which is associated with glycolysis upregulation, as an optimal condition for viral replication. Moreover, hypoxia-triggered metabolic reprogramming, mediated by ROS induction and HIF activation, is exploited by DENV for further increase of its replication.

## 5. Conclusions

In summary, we report that low oxygen tension selectively promotes DENV RNA replication in cultured cells. This enhancement is HIF and AKT dependent and correlates with an increase in anaerobic glycolysis that elevates ATP production. Although hypoxia enhances replication of both DENV and HCV, the dependency on HIF for this enhancement differs fundamentally, which might be a reflection of the different metabolic requirements of these two members of the *Flaviviridae* family. Moreover, we note that DENV can induce hypoxic response and subsequent metabolic shift in host cell, in an AKT-independent but ROS-dependent manner.

Thus, this study opens new possibilities in defining important metabolic determinants of DENV replication and defining novel therapeutic targets.

## Supplementary Materials

The following are available online at http://www.mdpi.com/2073-4409/7/12/241/s1. Figure S1: DENV replication kinetics at 20% O_2_—Effect of cell confluence on DENV replication, intracellular ATP levels, and HRE-Luc activity, Figure S2: DENV RNA replication and intracellular ATP is enhanced in hypoxic immortalized hepatocytes, monocytes and epithelial cells, Figure S3: Chemical hypoxia in DENV-infected cells, Figure S4: Effect of PI3K inhibition on oxygen-regulated increase of DENV replication, Figure S5: Redox homeostasis and DENV infection.
