# Supplementary material for: The Role of Tissue Oxygen Tension in Dengue Virus Replication
Source: Cells. 2018 Dec 1;7(12):241. doi: 10.3390/cells7120241 (PMC6316080; doi:10.3390/cells7120241)
Supplement: Supplementary file 1 [file cells-07-00241-s001.pdf]

cells

MDPI

Article

The Role of Tissue Oxygen Tension in Dengue Virus Replication

Efseveia Frakolaki [1], Panagiota Kaimou [1], Maria Moraiti [1], Katerina I. Kalliampakou [1], Kalliopi Karampetsou [2], Eleni Dotsika [2], Panagiotis Liakos [3], Dido Vassilacopoulou [4], Penelope Mavromara [5], Ralf Bartenschlager [6,7] and Niki Vassilaki [1,] *

1 Laboratory of Molecular Virology, Hellenic Pasteur Institute (HPI), 11521 Athens, Greece; sevif@pasteur.gr (E.F.), yioula.kai27@gmail.com (P.K.); moraiti.biology@gmail.com (M.M.); e.kalliampakou@pasteur.gr (K.I.K.)
2 Laboratory of Cellular Immunology, Hellenic Pasteur Institute, 11521 Athens, Greece; karampetsou@pasteur.gr (K.K.); e.dotsika@pasteur.gr (E.D.)
3 Laboratory of Biochemistry, School of Medicine, University of Thessaly, 41500 Larissa, Greece; pliakos@med.uth.gr
4 Section of Biochemistry and Molecular Biology, Faculty of Biology, National and Kapodistrian University of Athens, 15701 Athens, Greece; didovass@biol.uoa.gr
5 Laboratory of Biochemistry and Molecular Virology, Department of Molecular Biology and Genetics, Democritus University of Thrace, 68100 Thrace, Greece; pmavrom@mbg.duth.gr
6 Department of Infectious Diseases, Molecular Virology, University of Heidelberg, 69120 Heidelberg, Germany; Ralf.Bartenschlager@med.uni-heidelberg.de
7 German Center for Infection Research, Heidelberg partner site, 69120 Heidelberg, Germany.
* Correspondence: nikiv@pasteur.gr; Tel.: +30-210-647-8875

Supplementary Materials and Methods

Cell Culture

Immortalized Human Hepatocytes (IHH) (originally obtained from ATCC) [1] were cultured in high glucose (25 mM) Dulbecco's modified minimal essential medium (Thermo Fisher Scientific, Waltham, Massachusetts, USA), supplemented with 2 mM L-glutamine, 0.1 mM non-essential amino acids, 100 U/mL penicillin, 100 µg/mL streptomycin and 10% (v/v) fetal calf serum (referred to as complete DMEM). THP-1 monocytic cells (kindly provided by E. Meurs, Institute Pasteur, Paris, France) [2] were cultured in RPMI-1640 supplemented with 2 mM L-glutamine, 100 U/mL penicillin, 100 µg/mL streptomycin and 10% (v/v) fetal calf serum.

ROS Quantification

DENV-infected and non-infected Huh7 cells were probed with 5 µM carboxy-H2DCFDA (Thermo Fisher Scientific, Waltham, Massachusetts, USA) for 30 min at 37 °C and 5% CO_2 atmosphere. As positive control, non-infected cells were treated with 1 µg/ml LPS for 15 min, before incubation with the probe. 10,000 cells per experimental group were analyzed for intracellular ROS with FACS Calibur Cytometer (Becton-Dickinson, Franklin Lakes, NJ, USA) and data were analyzed with FlowJo V.10.0.8 software (Tree Star Inc, Ashland, OR, USA) to calculate the Geometric Mean of fluorescence intensity.

Chemicals

LY294002 (panPI3K iinhibitor) was obtained from Cayman Chemical (Ann Arbor, Michigan, USA). H_2O_2 was purchased by Sigma-Aldrich (Taufkirchen, Germany).

Supplementary Data

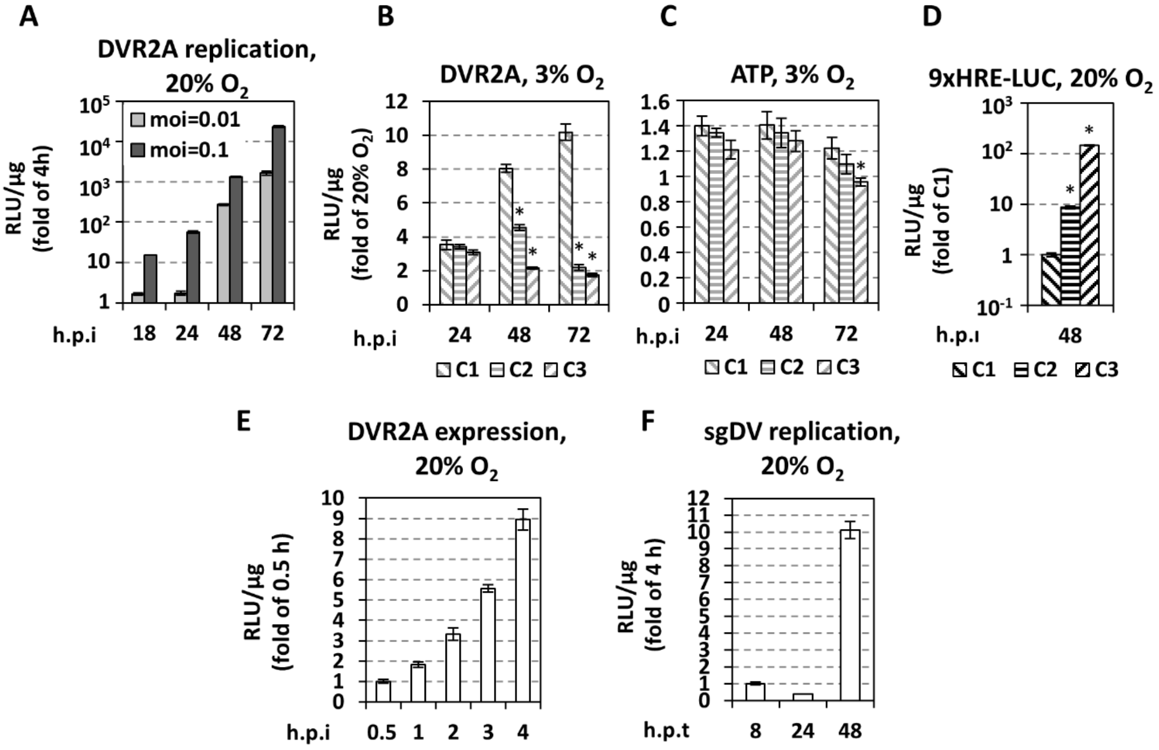

Figure S1. DENV replication kinetics at 20% O_2 - Effect of cell confluence on DENV replication, intracellular ATP levels and HRE-Luc activity. (**A**) DVR2A replication kinetics in Huh7 infected cells (MOI 0.01 or 0.1) at 20% O_2 (Figure 2C). Cells were inoculated with DVR2A for 4 h and lysed at indicated h p.i. DVR2A-derived R-Luc activity was measured and expressed as RLU/μg of total protein amount. Values are expressed as fold of the ones obtained at 4 h. (**B**–**D**) Cell density affects DENV replication, HRE activation and ATP levels. Huh7 cells were seeded in three different concentrations (C1, C2, C3) representing 30%, 60% and 90% cell confluence, respectively. After preincubation at 20% (B-D) or 3% O_2 (B-C) for 18 hours, cells were either infected with DVR2A (MOI 0.01), or transfected with 9xHRE-Luc construct. DVR2A R-Luc activity and intracellular ATP levels from infected cells, as well as HRE F-Luc activity, were measured and expressed as RLU/μg of total protein amount. Values from cells cultured at 3% O_2 are expressed as fold of the ones under 20% O_2 (B-C). For HRE-transfected cells values are expressed as fold of C1 confluency. Panels B,D: * $p < 0.001$ vs 20% O_2 cells. Panel C: * $p < 0.01$ vs. 20% O_2 cells (Student's t test). (**E**) DVR2A early replication kinetics in Huh7 infected cells (MOI 1) at 20% O_2 (Figure 3A). Cells were inoculated with DVR2A and lysed at indicated h p.i. DVR2A-derived R-Luc activity was measured and expressed as RLU/μg of total protein amount. Values are expressed as fold of the ones obtained at 0.5 h. (**F**) Viral RNA replication at 20% O_2 (Figure 3C), as determined by measuring R-Luc activity upon DENV RNA electroporation with 5 μg RNA/4 × 10^6 cells subgenomic sgDVR2A (sgDV). Error bars in all panels represent SD of at least two experiments in triplicates.

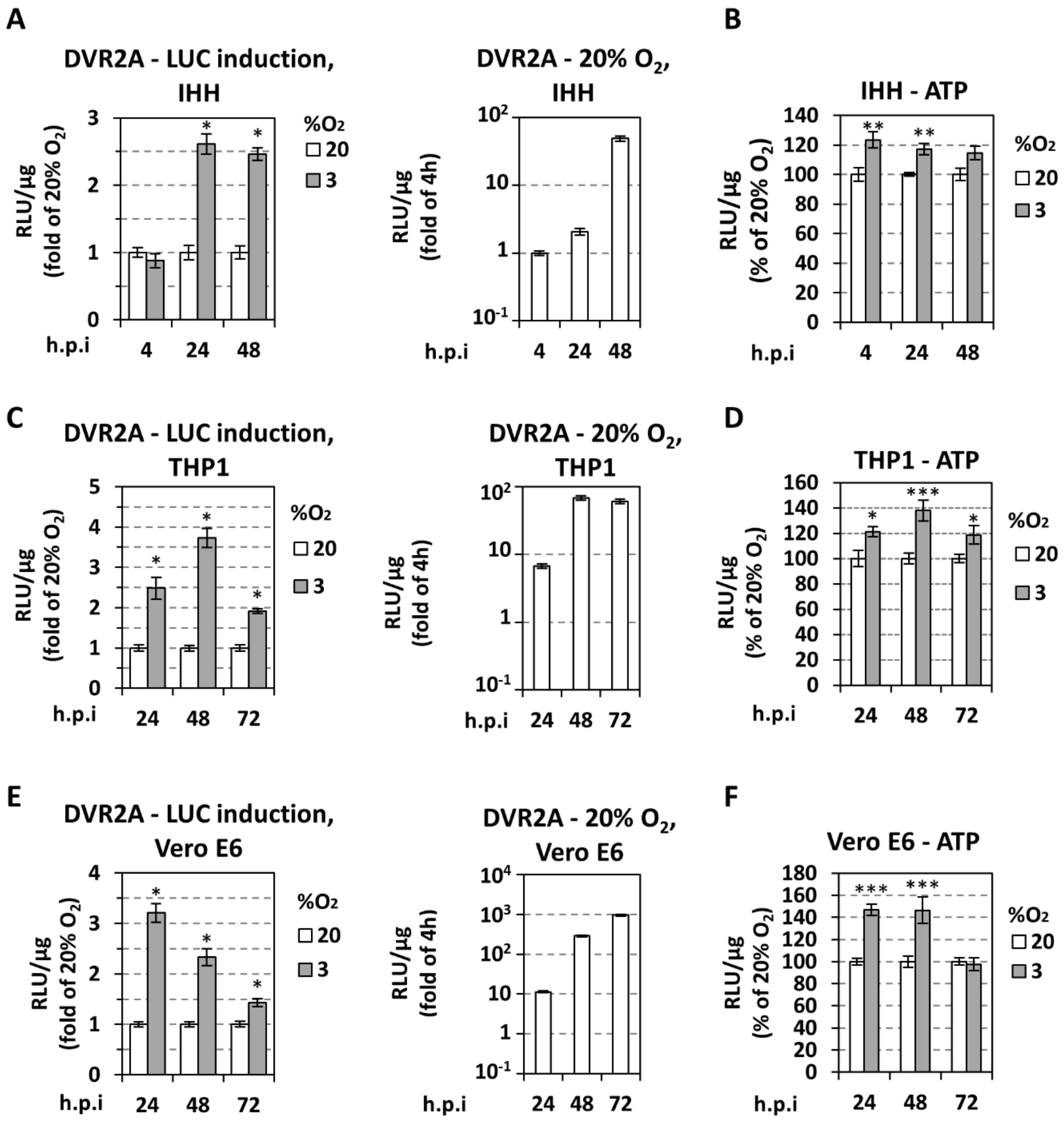

Figure S2. DENV RNA replication and intacellular ATP is enhanced in hypoxic immortalized hepatocytes, monocytes and epithelial cells. Cells preincubated at 20% or 3% v/v O_2 were inoculated for 4 h with DVR2A at MOI = 0.1 for IHH and THP1 cells or MOI 0.01 for Vero E6 and further incubated at the preincubation conditions until harvest. DVR2A replication-reflecting luciferase activity (**A**,**B**) and intracellular ATP levels (**C**) were measured and are expressed as RLU/μg of total protein amount. (**A**) Fold difference of luciferase levels obtained with cells incubated at 3% versus 20% O_2. (**B**) Replication kinetics at 20% O_2. Values are expressed as fold of the ones obtained at 4 h. (**C**) ATP values from cells cultured at 20% O_2 at each time-point were set to 100. Bars represent mean values from at least three independent experiments in triplicate. Error bars indicate standard deviations. Panels A,C,E: * $p < 0.001$ vs. 20% O_2 cells. Panels B,D,F: * $p < 0.05$, ** $p < 0.01$, *** $p < 0.001$ vs 20% O_2 cells (Student's t test).

A

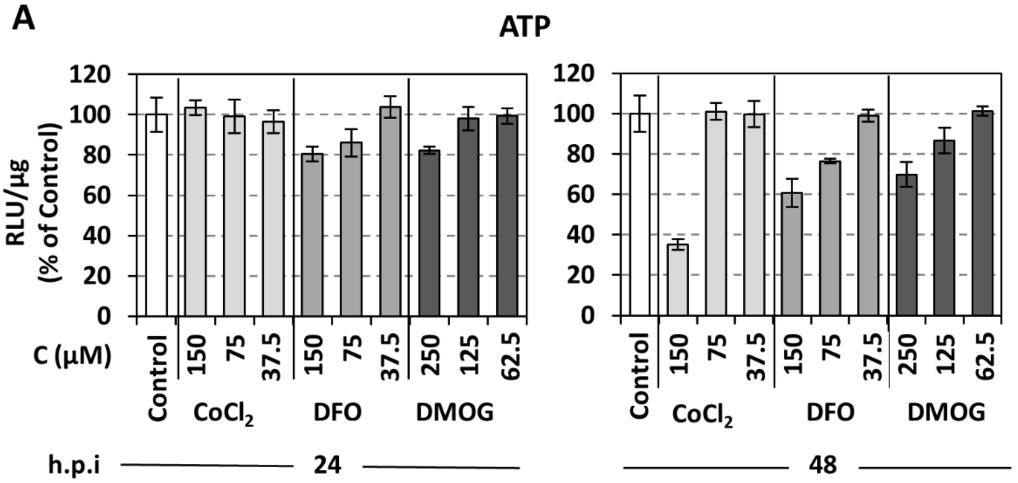

B

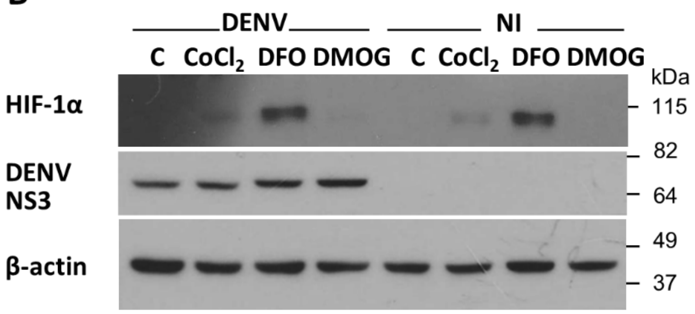

Figure S3. Chemical hypoxia in DENV-infected cells. (**A**) Intracellular ATP levels in Huh7 cells infected with DVR2A (MOI 0.01). 4 h post virus inoculation cells were treated with the indicated concentrations of $CoCl_2$, DFO or DMOG. Values (RLU/µg of total protein) are expressed as percentage of the reporter activity derived from control-non treated cells. (**B**) Lower intensity version of the chemiluminescent Western blot image of Figure 6C.

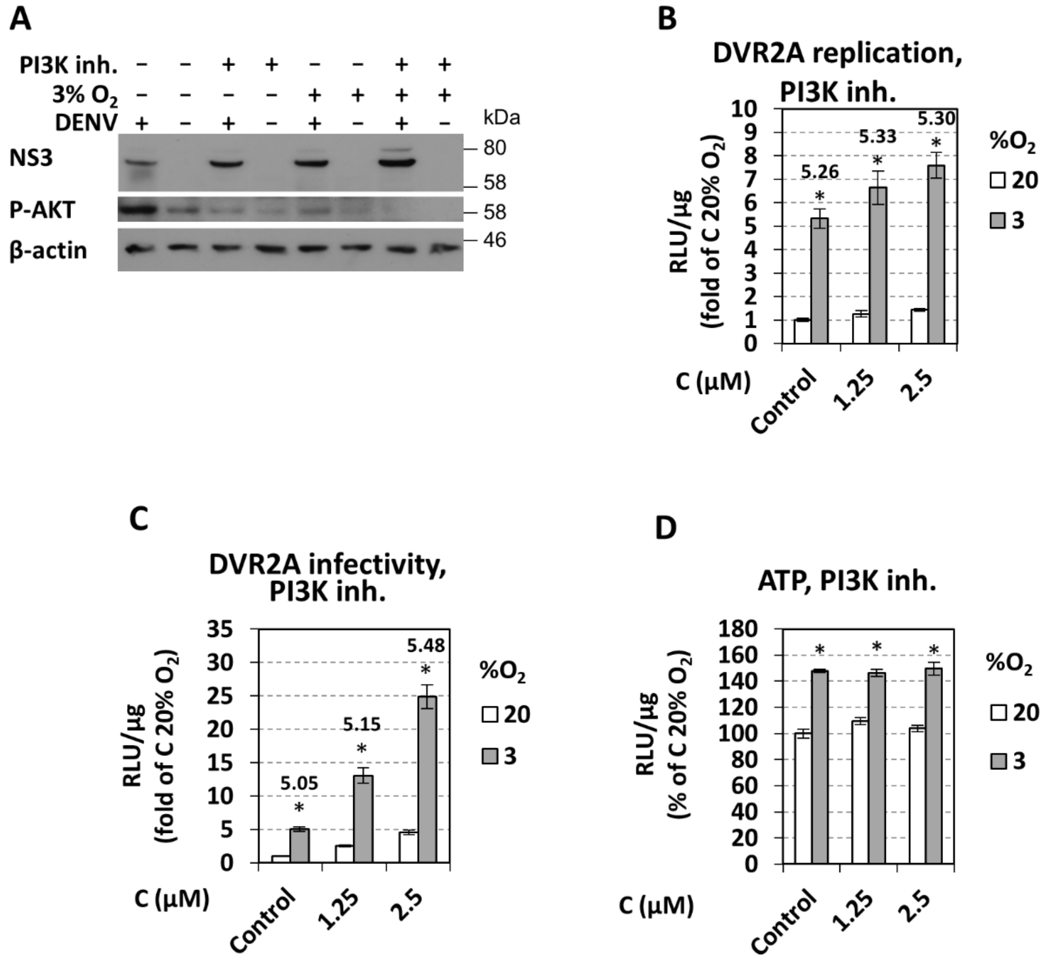

Figure S4. Effect of PI3K inhibition on oxygen-regulated increase of DENV replication. (**A-D**) Huh7 cells, preincubated at 3% or 20% O_2 for 18 h, were infected with DENV (MOI 0.5) or DVR2A (MOI 0.1). Non-infected cells were used as control. After virus inoculation, PI3K inhibitor LY294002 was added at the specified concentrations and cells were further incubated at the pre-incubation conditions. (**A**) Western blot analysis of DENV NS3 (top), P-AKT (middle) and β-actin (bottom). β-actin served as a loading control. A representative experiment of 3 independent repetitions is shown. (**B**) DVR2A-derived R-Luc activity (RLU/μg of total protein) from infected cells. Values obtained from control-DMSO treated cells incubated at 20% O_2 were set to one. Fold difference of values measured at 3% O_2 versus the corresponding ones at 20% O_2 are depicted on the top of the bars. (**C**) Release of infectivity of Huh7 cells, previously infected with DVR2A at MOI 0.1 at the indicated oxygen conditions and treated with LY294002. Supernatants from these cells were collected at 48 h p.i. and used to infect naïve Huh7 cells (infected and incubated at 20% O_2), 72 h post-infection the cells were lysed and the luciferase activity was measured and expressed as RLU/μg of total protein amount. Values obtained using supernatants of control-DMSO treated cells incubated at 20% O_2 were set to one. Fold difference of values measured at 3% O_2 versus the corresponding ones at 20% O_2 are depicted on the top of the bars. (**D**) Intracellular ATP levels (RLU/μg of total protein) from infected cells. Values obtained from control-DMSO treated cells incubated at 20% O_2 were set to 100. In all panels, bars represent mean values from at least three independent experiments in triplicate. Error bars indicate standard deviations. * $p < 0.001$ vs. 20% O_2 cells (Student's t test).

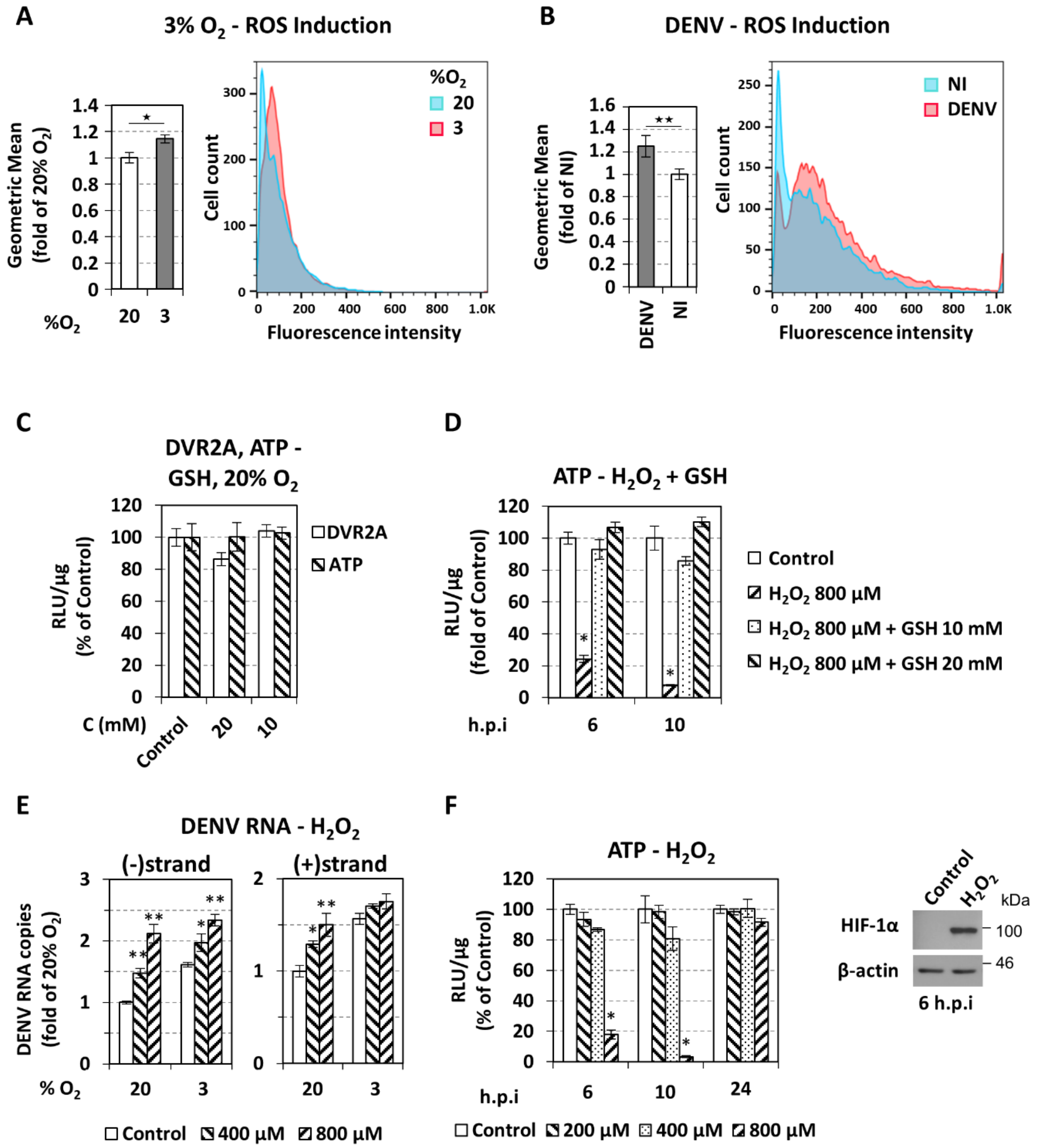

Figure S5. Redox homeostasis and DENV infection. (**A**) **Induction of ROS production in hypoxic cells.** Ratio of geometric mean intensity (left) between levels of ROS generation in Huh7 cells incubated at 3% versus 20% O_2 for 48 h, and the respective overlay histogram (right). ROS production in both oxygen conditions was determined by FACS analysis (H2DCFDA fluorescence). (**B**) **Induction of ROS production upon DENV infection.** Ratio of geometric mean intensity (left) between levels of ROS generation in DENV-infected versus non-infected Huh7 cells incubated at 20% O_2 for 48 h, and the respective overlay histogram (right). ROS production in both conditions was determined by FACS analysis (H2DCFDA fluorescence). For the geometric mean, the average from three experiments for each condition is shown. For the overlay histograms, a representative experiment of 3 independent repetitions is shown. * $p < 0.01$ vs 20% O_2 cells, ** $p < 0.001$ vs. NI cells (Student's t test). (**C**,**D**) **Effect of glutathione on DENV replication and ATP levels at 20% O_2**. (**C**) Huh7 cells were infected with DVR2A (MOI 0.01) at 20% O_2. After virus inoculation, reduced glutathione (GSH) was added at the specified concentrations and cells were lysed at 48 h p.i. DVR2A-derived R-Luc activity and intracellular ATP levels were measured and expressed as RLU/µg of total protein. Values are expressed as percentage of the control-not treated cells. (**D**) Huh7 cells, preincubated at 3% or 20% O_2 for 18 h, were infected with DENV (MOI 0.5). 2 h after virus inoculation, H_2O_2, or a combination of

H_2O_2 and reduced glutathione (GSH) were added at the specified concentrations. Cells were further incubated at the pre-incubation conditions and medium was renewed every 4 h, up to 10 h p.i. Intracellular ATP levels were measured and expressed as RLU/μg of total protein. Values obtained from control-non treated cells were set each time to 100. *p<0.001 vs control cells. (**E**,**F**) **Effect of H_2O_2 on intracellular ATP and DENV RNA levels.** Huh7 cells, preincubated at 3% or 20% O_2 for 18 h, were infected with DENV (MOI 0.5). 2 h after virus inoculation, H_2O_2, or a combination of H_2O_2 and reduced glutathione (GSH) were added at the specified concentrations. Cells were further incubated at the pre-incubation conditions and medium was renewed every 4 h, up to 10 h p.i. H_2O_2 was replenished in order to maintain the level of H_2O_2 in cell culture, which is known to be rapidly reduced due to the activity of cellular degrading enzymes, primarily catalase, and the intracellular production of glutathione. (**E**) qRT-PCR analysis of intracellular DENV-negative (−) and -positive (+) strand RNA copies from Huh7 cells at 10 h p.i. Values are expressed relative to the ones from non-treated cells at 20% O_2. * $p < 0.01$, ** $p < 0.001$ vs control cells. (**F**,**left**) Effect of H_2O_2 on intracellular ATP in the presence or absence of GSH at 20% O_2. Values obtained from control-non treated cells were set each time to 100. * $p < 0.001$ vs control cells. (**F**,**right**) Western blot analysis of HIF-1α (top) and β-actin (bottom) in cells treated or not with 800 μM H_2O_2. β-actin served as a loading control. A representative experiment of 3 independent repetitions is shown.

References

1. Lasfer, M.; Vadrot, N.; Aoudjehane, L.; Conti, F.; Bringuier, A.F.; Feldmann, G.; Reyl-Desmars, F. Cadmium induces mitochondria-dependent apoptosis of normal human hepatocytes. *Cell biology and toxicology* **2008**, *24*, 55-62, doi:10.1007/s10565-007-9015-0.
2. Tsuchiya, S.; Yamabe, M.; Yamaguchi, Y.; Kobayashi, Y.; Konno, T.; Tada, K. Establishment and characterization of a human acute monocytic leukemia cell line (THP-1). *International journal of cancer* **1980**, *26*, 171-176.
